# Comparative evaluation of the apical sealing ability of a ceramic based sealer and MTA as root-end filling materials – An *in-vitro* study

**DOI:** 10.4317/jced.53903

**Published:** 2017-07-01

**Authors:** Bhavana Gandhi, Ramesh Halebathi-Gowdra

**Affiliations:** 1 Assistant Professor, Department of Conservative dentistry and Endodontics, Siddhartha Institute of Dental sciences, Gannavaram, Andhra Pradesh; 2 Professor, Department of Conservative dentistry and Endodontics, Al Qaseem private colleges, College of dentistry, Buraydah, Kingdom of Saudi Arabia

## Abstract

**Background:**

The present study was aimed to evaluate and compare the apical sealing ability of two endodontic root-end filling materials namely, iRoot SP (ceramic based) and ProRoot MTA using the bacterial leakage system.

**Material and Methods:**

A total of fifty recently extracted, single rooted teeth with a single straight canal were selected for the study. The teeth were chemo mechanically prepared. The apical 3mm of the root was resected and root end cavities were prepared. The teeth were randomly divided into two groups of twenty teeth each for the experimental root end filling materials namely, iRoot SP and ProRoot MTA. A two-chamber model was constructed using pippeter tips and plastic vials. The pipetter tips with the teeth were suspended in these caps and the entire assembly was reattached to the vial. The upper chamber was seeded with*Enterococcus faecalis*. An Enterococci-selective broth was used in the lower chamber. Leakage was assessed for 90 days and compared using survival statistics.

**Results:**

The ProRoot MTA filled root end samples leaked within 30-72 days. The iRoot SP filled root end samples leaked within 51-69 days.

**Conclusions:**

Under the parameters of this study, it can be concluded that all the tested materials showed significant apical sealing ability as root-end filling materials over a period of 90 days. iRoot SP exhibited the most effective apical sealing ability as compared to ProRoot MTA.

** Key words:**Apical sealing ability, Bacterial leakage, iRoot SP, ProRoot MTA, Root-end filling.

## Introduction

The best result visualized for an endodontic treatment would be hard tissue closure, separating the obturated canal from the periapical tissues and maintaining a biologically conducive environment (1). Among many causes leading to endodontic failure, microleakage between the root canal filling material and the root canal walls adversely affects endodontic prognosis (2). So it is imperative that complete sealing of the root canal system following biomechanical preparation is carried out to prevent re-infection of the canal and periapical tissues (3). An endodontic sealer fulfils this role by filling the spaces of the root canal system and also bonds the core material to the dentinal walls (4). An ideal root canal sealer should be biocompatible, antibacterial, nontoxic, radiopaque, provide a fluid impervious seal, dimensionally stable, and have a good adhesion to the root canal wall (5).

The dynamic metabolic processes in the periapical tissues make the filling of the apical third of the root canal different from the rest of the canal. The property of osseoconductivity in an endodontic sealer helps in achieving three dimensional closure of root canal orifice in a wet environment in time (6). A root end filling material is basically used to improve the seal of the periapex (6).

A plethora of materials have been proposed as retrograde filling materials but in recent years, Mineral Trioxide Aggregate (MTA) has received tremendous popularity among all (7). A new obturation sealer, iRoot SP (Injectable Root canal Sealing Paste, Verio Dental, Vancouver, Canada), has recently been introduced to the market. According to the manufacturer’s description, iRoot SP is a convenient, premixed, ready-to-use injectable white hydraulic cement paste developed for permanent root canal filling, as an endodontic sealer and as a retrograde filling material. iRoot SP is an insoluble, radiopaque, and aluminium-free material based on a calcium silicate composition, which requires the presence of water to set and harden. To date there appear to be few studies evaluating the apical sealing ability of this new material (8).

Considering the successful outcomes observed with the use of this material, the present study was undertaken to compare and evaluate the apical sealing ability using the bacterial leakage system of two root-end filling materials namely: iRoot SP and Pro-Root MTA (DENTSPLY).

## Material and Methods

-Preparation of the teeth

Fifty intact freshly extracted single rooted permanent teeth with complete root formation were collected and stored in 0.5% sodium hypochlorite solution for three days to remove organic debris and disinfect the surface. Further all teeth were washed under tap water to remove sodium hypochlorite residues. Pre-operative radiographs were taken to confirm all the teeth were single rooted and had a single straight canal. All teeth were cleaned free of attached soft tissue and stored in normal saline solution until use.

Standard access cavities were prepared and a #15K file was used to establish apical patency. When the file tip appeared flush with the apical foramen, the length of the file was recorded; the working length was determined 1 mm short of the measured length. The root canal system was instrumented using a crown-down technique. The coronal portion of the canal was enlarged with Gates Glidden drills up to No.3 size and the rest of the canal using ProTaper Universal (DENTSPLY) nickel-titanium rotary instruments. To obtain a standardized apical diameter all samples were prepared to an ISO size 40. 3% sodium hypochlorite was used to irrigate the canals throughout the instrumentation procedure. To remove the smear layer formed after instrumentation 17% liquid EDTA for 1 min followed by 3% sodium hypochlorite was used along with final rinse of normal saline.

With continuous water spray the apical 3mm of the root was removed with a bur perpendicular to the long axis of the root. Root end cavities were prepared with an ultrasonic diamond coated tip using water coolant to a depth of 3mm. To provide an intracanal matrix to pack the root end filling material against, a flattened ISO size 70/0.02 gutta percha cone was inserted into the canal leaving a root-end void of 3mm. The root canals were dried with sterile paper points before the root-end fillings were placed.

-Root end obturation

The roots were randomly divided into two control groups of five teeth each and two experimental groups of 20 teeth each to receive ceramic based sealer – iRoot SP and ProRoot MTA as a root end filling material. To prevent bacterial leakage through the root surfaces, two layers of nail varnish were applied to the external surfaces of all roots, except to the resected root ends and the root-end filling materials. Following the placement of the intracanal barrier in the root canals of the control teeth, five root-end cavities were filled with gutta-percha without a root canal sealer which was a positive control and the other five were filled with sticky wax covered with two layers of nail varnish which would act as a negative control. The samples were then wrapped in gauze saturated with sterile saline until use.

-Apparatus set-up

**Figure 1:**
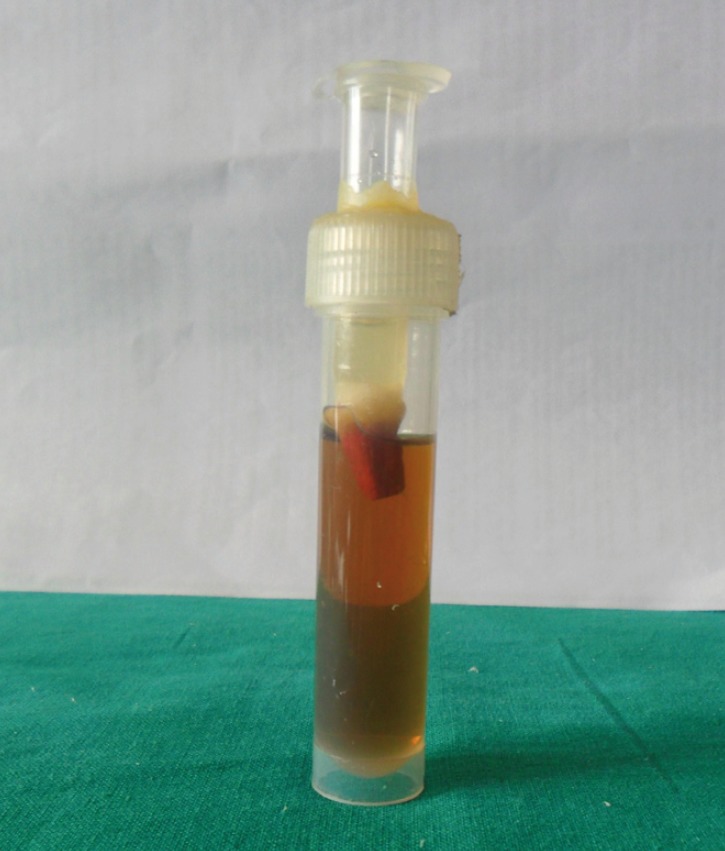
Pipetter tips with inserted teeth snugly fitted into the holes of the lids and reattached to the vials; Broth placed in the lower chamber.

An experimental system similar to that suggested by Barthel *et al.* (9-12) was used. 2.7 ml air tight Micro Centrifuge Tube-D with attached hinged cap and autoclavable capacity, were cut off at the narrow end individually for each specimen so that the rim fitted 1 mm apically from the root equator. Subsequently, the root was sealed in the pipette tip using sticky wax covered by two layers of nail varnish, so that the most apical 3 mm remained free of the sealant. Twelve ml plastic vials with snap-on plastic caps were used to suspend the prepared teeth in a broth compatible with *E. faecalis*. A round bur with a diameter of 9 mm was used to bore a hole through the centre of each cap. Pipetter tips with inserted teeth were snugly fitted into the holes of the lids, so that the tooth root extended into the vial (Fig. 1). Pipetter tips were sealed to the lids using sticky wax and the joint was sealed with two layers of nail varnish.

The prepared teeth along with their caps and the vials were sterilized overnight with ethylene oxide gas and then aerated for two days in a sterile environment. After autoclaving, the glass vials were filled with the broth to a level of 3 mm above the resected root end. The lids containing the pipetter tips and the teeth were reattached to the glass vials.

Bacterial Preparation

Using a sterile micropipette a 0.1 ml of overnight broth culture of *Enterococcus faecalis*, was inoculated into the root canal of each tooth via the coronal access cavity preparation. The opening of the pipette tips was covered with sterile plastic caps to impede evaporation and prevent contamination. Penetration of the *Enterococcus faecalis* from the root canals into the broth will result in formation of an acid and a change of colour in the indicator solution indicating bacterial leakage. The entire apparatus was then placed into an incubator maintained at a constant 37˚C. All the procedures were performed under aseptic technique.

-Monitoring the samples

**Figure 2:**
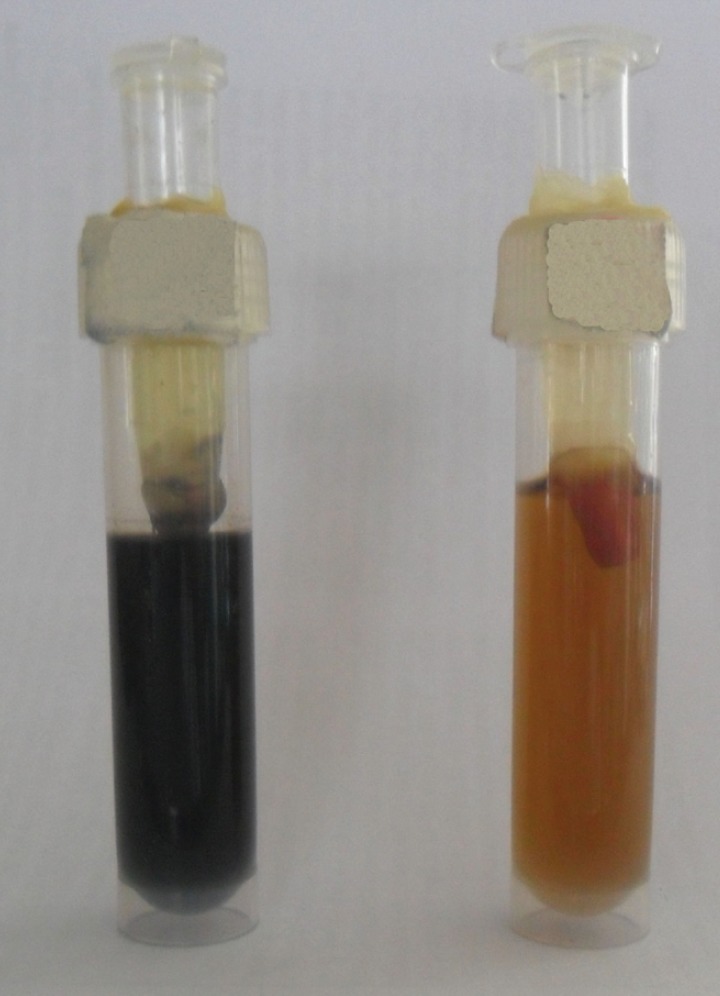
Change of colour in the lower chamber in the left sample indicating bacterial leakage.

To ensure the viability of *Enterococcus faecalis*, fresh overnight culture of the organism was added to the root canals every alternate day after removal of the old culture. The broth was monitored for color change (Fig. 2) daily up to 90 days which was the experimental time for this study. As and when there was a color change in the samples, a sample of the medium was placed on blood agar to ensure that it contained the same type of bacteria as that placed in the tubing.

-Statistical analysis

**Figure 3:**
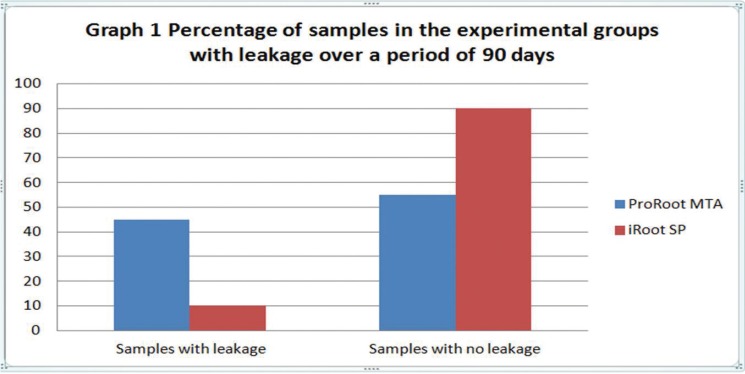
Comparison of the percentage of samples in the experimental groups with leakage over a period of 90 days.

The duration until a specimen leaked (in days) was recorded as event time graph (Fig. 3) for a period of 90 days and the results were evaluated and compared using Chi-square test and Mann Whitney test. The *p* value was < 0.05 and therefore was considered statistically significant.

## Results

All the samples in the positive controls showed leakage. Among the negative controls 4 out of 5 samples did not show leakage. The ProRoot MTA filled root end samples leaked within 30-72 days with the mean being 56.6 days, median being 67 and Standard Deviation being 17.91. The iRoot SP filled root end samples leaked within 51-69 days with the mean being 60 days, median being 60 and Standard Deviation being 12.72 (Fig. 3).

## Discussion

Endodontic success attributes to several factors associated at every stage of treatment. A case might appear simple but unless it is meticulously performed may lead to failure. The various causes associated with endodontic failure using conventional method of root canal treatment include incomplete chemo-mechanical debridement, persistence of bacteria in the canals and apex, poor obturation quality, over and under extension of the root canal filling, coronal leakage, perforations, instrument breakage, calcifications and anatomic anomalies (9,10). A root end filling material is indicated when there is an open apex, blunderbuss canal, root resorption or when a conventional root canal treatment has failed and a surgical approach is required. 10 Among the various requirements of a root-end filling material, a biologically conducive fluid impervious seal apically is of utmost importance because the periapical environment is dynamic (6).

Almost every restorative material available has been tried as a root-end filling material (11). The various materials tested in recent studies as a root-end filling material *in-vitro* include MTA, amalgam, IRM, retroplast, Geristore, Super-EBA, Titanium, Diaket, GIC, N-Rickert, Gutta Percha, Resilon, 4-META/MMB-TBB resin (SuperBond), CYMED 8410, Bioaggregate, Polymer nano-composite (PNC) resins, ERRM putty, ERRM paste, and iRoot BP (11) iRoot SP Root Canal Sealer, is an aluminium-free sealer based on a calcium silicate composition. According to the manufacturer, iRoot SP is composed of biocompatible and nontoxic materials that include calcium phosphate, calcium silicates, zirconium oxide, and calcium hydroxide. iRoot SP includes a similar composition to white mineral trioxide aggregate (MTA) material and has both excellent physical properties and biocompatibility. iRoot SP can form a hermetic seal inside the root canal and be used for filling root canals with or without gutta-percha points to form a monoblock (8). A PubMed search on iRoot SP, shows 27 studies on it till date with only two testing its apical sealing ability. Therefore this study was undertaken to evaluate and compare the apical sealing ability of iRoot SP and ProRoot MTA.

Apical leakage is the infiltration of the apical root segment by peptides and other molecules, helping the microbes to survive in an obturated root canal (12). Apical leakage has been the area of research for every dental material available and a number of methods have been utilized with differing success rates in their methodology. The bacterial leakage model has been chosen as the method of choice in the determination of apical leakage in the present study as bacteria serve as biological markers of assessing leakage (13-16). Previous studies have shown that the bacterial leakage model proved suitable for testing the apical sealing ability. It provides data that are more biologically significant and clinically relevant than other methods (13-15). There are three methods available for assessment of bacterial microleakage, namely, the dual-chamber leakage model, detection of bacteria using a scanning electron microscope (SEM), and polymerase chain reaction (PCR) (14,15).

In the dual-chambered or the split-chambered model, the tooth to be tested is sealed in between the upper and lower chamber (15). Turbidity or a color change in the sterile broth in the lower chamber will show the microleakage of viable microorganisms from the upper chamber (14,15) The model used in this study was patterned after that designed by Torabinejad *et al.* (17-19). In the upper chamber, a nonselective, non discolouring broth was chosen to prevent false positive results from the leakage of dye molecules rather than bacteria. Purity of growth was checked throughout the experiment (10,12).

The dual-chamber model, may contain a single species (*E. faecalis, S. mutans, P. mirabilis, or S. epidermidis*), multiple species, or saliva in the upper chamber (15). *E. faecalis* was chosen as the microbial marker as it has been identified as the species most commonly found in root canals of teeth presenting failure after endodontic treatment thus improving the clinical relevance of this study. It can be found in root canals submitted to conventional endodontic treatment owing to their ability to resist antimicrobial agents used for root canal irrigation and calcium hydroxide root canal dressing, because they are able to survive at extremely high pH (10,11,13).

In the present study an infection period of 90 days was allowed to ensure adequate penetration of the organism through the root end into the broth. A single microorganism was used to contaminate the root canals to allow for ease of maintaining a single species. The interface between the upper and lower chamber was completely eliminated in order to provide only one route for micro-leakage: through the tested obturated material. This was done through the placement of growth medium broth containing *E. faecalis* directly in the access cavity of the experimental teeth (14,15). The success of the negative controls in showing no leakage and the failure of positive controls in showing any leakage has validated the successful experimental design of the apparatus.

The color change in nearly all the samples in the positive control group indicates that root canal sealer is needed to improve gutta-percha as root canal filling material, as shown by Becker and Von Fraunhofer (15) with thermoplasticized gutta-percha with and without root canal sealer.

Following the completion of the study, there were a plethora of things to be learnt from it. To begin with, an experimental study was carried out by Rechenberg *et al.* (13) to evaluate whether published studies on microbial leakage through filled root canals in human teeth embedded in a two-chamber system were properly controlled. They concluded that the methods under evaluation appeared unsuitable for comparing different permanent root filling materials at this point. Future studies should aim at working on this aspect and involve proper controls and histology with the help of Confocal laser scanning microscopy (13). Another fact was that it is not possible to estimate the time of occurrence of periradicular infection using microbial leakage studies because it depends on factors such as the virulence of microorganisms, defense capacity of the periradicular tissues, nutritional status, and bacterial interactions. But, chronic or acute infections may occur when microorganisms are present at the periapex (14).

Every leakage study methodology (dye penetration, fluid filtration, or electrochemical tests) has its limitations so does the dual chambered model utilized in the present study. There are multiple routes of bacterial entry via the dual chambered leakage model with sticky wax being one of them. In a study done by Rechenberg *et al.* (13), it was observed that a blackened cuff was present between the sticky wax and the cementum of the experimental tooth as seen during tooth sectioning, which was also seen in the present study specimens. This indicates that sticky wax is a poor sealant and a better material is required to serve its purpose.

To combat the limitations of the dual chambered model, and better control the factors involved, modifications have been proposed (13) in the construction of the model. These include, the apices of the teeth can be covered with epoxy resin rather than sticky wax, an enterococci-selective broth can be used in the lower chamber to avoid contamination, in the upper chamber, a nonselective, nondiscolouring broth can be chosen to prevent false positive results, a DNA stain (Syto59) that is actively transported into the cell and thus almost exclusively stains viable cells can be used to trace bacterial leakage, and root transsections rather than longitudinal sections can be used to trace the presence of viable bacteria (13).

On evaluation of the apical sealing ability of the ceramic based sealer and MTA based sealer, all the tested materials showed significant difference in their apical sealing ability as root end filling materials. In addition the apical sealing ability of iRoot SP proved to be more efficient than ProRoot MTA. Similar findings were observed by Zhang *et al.* (8).

## Conclusions

Under the parameters of the study, we can conclude that all the tested materials showed statistically significant apical sealing ability. Considering the properties of iRoot SP, it can be suggested that it proved to be a suitable material to be used as a root-end filling material. All the samples should be tested histologically using Confocal Laser microscopy and fluorescence to better validate the results.
